# Comparison of Aquatic Therapy vs. Dry Land Therapy to Improve Mobility of Chronic Stroke Patients

**DOI:** 10.3390/ijerph17134728

**Published:** 2020-07-01

**Authors:** Sagrario Pérez-de la Cruz

**Affiliations:** Department of Nursing, Physiotherapy and Medicine, University of Almería, 04120 Almería, Spain; spd205@ual.es; Tel.: +34-950-214-574

**Keywords:** aquatic therapy, gait, pain, stroke

## Abstract

One of the most serious and disabling problems of stroke is pain and a decrease in balance, with the consequent increased risk of falls. The aim of the randomized controlled trial study was to compare the efficacy of three different treatment proposals to improve pain, gait, and balance in chronic stroke patients. Forty patients diagnosed with stroke were divided into three groups: the dry-land therapy group (control group) received sessions that included walking exercises and trunk mobility. The experimental group received Ai Chi aquatic therapy, and the combined group received alternating dry-land therapy sessions and Ai Chi aquatic therapy. The measurement instruments used were: the Tinetti balance and gait scale, the visual analog scale (VAS), 360° turn, single leg stance, and the 30-s stand test (CS-30). After twelve weeks of treatment, the results were significantly better for the combined therapy group and the experimental group compared to the dry-land therapy group (*p* < 0.01) in the VAS scale, CS-30, and 360° turn, although improvements were also found in the evaluations carried out in the aquatic therapy group. In total, for the Tinetti scale and single-leg stance, the differences between the groups were evident, although not statistically significant (*p* = 0.001). Aquatic therapy with Ai Chi and the combination of aquatic therapy with dry-land therapy was effective in improving pain, balance, and gait in patients with chronic stroke, thus improving their functional capacity and quality of life.

## 1. Introduction

A stroke is a blood vessel disorder that leads to an acute loss of brain function for 24 h or more. According to the World Health Organization, about 15 million people have a stroke each year, and 5 million will be permanently disabled by it [1,2].

Among the serious neurological deficits caused by a stroke, the most significant is the loss of motor functions, as well as induced paralysis, pathological reflexes, and spasticity, which interfere with a person’s independent mobility [3,4]. One of the main consequences of this motor loss is hemiparetic gait, characterized by asymmetry in spatial-temporal parameters, with alteration of step length and stance phase. In addition, there is a reduction in the amplitude of hip and knee flexion, with an increase in plantar flexion or loss of dorsal flexion capacity at the level of the ankle [4,5]. This leads to a decline in balance with an increased risk of falls and limitations in the individual’s activities of daily living and participation in social activities in the community [6].

Rehabilitation after stroke has been shown to be of great importance in the recovery of function [7]. One of the available rehabilitation options is to perform the guided exercise in the water. Thus, the environmental characteristics of water influence physiological processes, motor activity, and spasticity, providing the patient with an enabling and motivating environment [8]. The essential physical properties of water are density, hydrostatic pressure, buoyancy, viscosity, and thermodynamics [9]. Buoyancy helps compensate for the gravity present in dry land and is, therefore, highly useful for therapy. Water viscosity is an exceptional source of natural resistance and can facilitate different motor training tasks [10] by providing resistance for muscle strengthening [11]. These characteristics of the aquatic environment allow some people with postural instability, high risk of falls, leg weakness, and gait disorders, be more successful when performing the exercise in an aquatic environment, compared to dry land [9].

Due to its ability to improve functional mobility while being enjoyable, water exercise has become a very popular form of physical training in the management of neurological disorders [10,11], being a safe environment and improving aspects of activity performance, quality of life and balance in people with brain damage [12,13]. In 2014, a systematic review on the effectiveness of aquatic therapy indicated that there is evidence that aquatic therapy improves dynamic balance and gait speed in people with neurological disorders, especially those with multiple sclerosis, Parkinson’s disease, and stroke [14]. Authors such as Chu et al. [15] have shown that stroke patients show improvements in gait speed after eight weeks of aquatic exercise. However, few studies have compared the effects of aquatic exercise over time (12 weeks) with land-based exercise and combined therapy in a group of patients diagnosed with non-traumatic acquired brain injury. The purpose of this study was to determine the effect of 12 weeks of treatment on pain, walking ability, and balance in patients with chronic stroke.

## 2. Materials and Methods

This study was a single-blinded, randomized controlled trial (NCT04168164). The study was conducted with individuals diagnosed with chronic stroke attending various associations in Spain between February–September 2018. The inclusion criteria for participation in the study were (1) patients with a minimum age of 35 years; (2) suffering a stroke, at least one year before the start of therapy; (3) the ability to move at least 10 m with the help of an assistive device or another person; (4) the ability to tolerate interventions and assessments; and (5) the ability to follow verbal commands. The exclusion criteria were (1) history of previous stroke or other adjuvant and degenerative neurological diseases (e.g., Parkinson’s disease and some type of dementia), (2) history of cardiovascular disorders, such as heart failure or arrhythmia, and (3) cognitive impairment identified by Mini-Mental State Examination (MMSE) <24.

**Figure 1:**
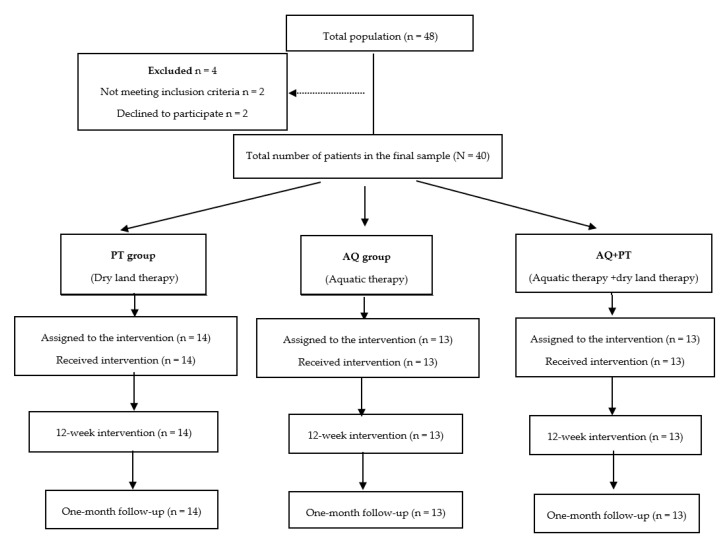
Study design flowchart.

All participants initially declared eligible to participate in this program, after being informed, gave their written consent, and the study was conducted in accordance with the regulatory standards of good clinical practice and the Declaration of Helsinki (2013), and approved by the Bioethics Committee of the University of Almeria (NCT04168164). The selection process of participants is shown in Figure 1.

The 40 patients who met the criteria were randomly divided into one of three groups (control group, experimental group, and combined group) using random numbers in sealed envelopes.

### 2.1. Assessment Tools

The Visual Analog Scale (VAS) is a continuous, single-item scale for assessing pain intensity. It is a 10-cm line with endpoints labeled “no pain” on the left and “pain as severe as possible” on the right. The patient is asked to rate the intensity of their pain by placing a mark on the line between the two endpoints. The distance between the two endpoints is measured using a ruler to obtain the score [16].

The Tinetti test [17] evaluates balance and gait in two main categories with 16 items: the initial nine items concern balance and the following seven focus on gait. The sum of both values (balance and gait) indicates the final score for this measure. A total score of 18 or less shows a high risk of falling, a total score between 19–24 shows a moderate risk of falling, and a total score of 24 or more shows a low risk of falling.

As a balance test, the 360° turn test was used. During this test, subjects are asked to make a 360° turn while standing in place. The time taken to perform this action was evaluated. Subjects are always asked to turn in the same direction [18]. Single leg stance balance tests were also performed [19]. During this test, while one leg of the patient was positioned in knee flexion, the other leg was kept in extension, timing how long participants were able to remain standing on one leg (measured on both sides) in an upright standing position. Two attempts were allowed, and the time they were able to remain on one foot was recorded.

The 30-s Chair Stand test (CS-30) [20]: The 30-CST is a measure that assesses the functional strength of the lower extremities in older adults. It is part of the Fullerton Functional Fitness Test battery. This test involves recording the number of repetitions (getting in and out of a chair) that a person can complete in 30 s rather than the amount of time it takes to complete a predetermined number of repetitions. Thus, it is possible to evaluate a wide variety of skill levels with scores ranging from 0 for those who cannot complete a repetition to more than 20 for fitter individuals.

### 2.2. Intervention

Evaluations were conducted by a single evaluator at the beginning of the study and after the study period. Finally, subjects were reassessed up to four weeks later to complete the measurements. This person was blinded to avoid any bias.

None of the participants experienced any significant treatment-related adverse events, and all patients in the intervention groups were fully compliant with the intervention program. There were no dropouts during the study.

#### 2.2.1. Dry Land Therapy (Control Group)

Participants assigned to the dry land therapy group (14 patients) (control group) received 24 sessions twice a week in total, over a period of 12 weeks. These sessions consisted of supervised group training sessions that lasted 45–50 min each. They consisted of a 10-min warm-up that included walking exercises, trunk mobility, and exercises involving the upper and lower extremities. The central part of the sessions consisted of 30–40 min of strength training, aerobic, flexibility, and coordination exercises, both individually and in groups. Each session was conducted with a specific intensity target, and ended with a cool-down period, consisting of 10 min of functional exercises based on activities of daily living, balance exercises, facial muscle exercises, proprioceptive exercises, muscle relaxation and stretching.

#### 2.2.2. Aquatic Ai Chi (Experimental Group)

The experimental group (13 participants) participated in 45-min group sessions, twice a week, for 12 weeks. The Ai-Chi therapy was performed by a physical therapist experienced in neurological rehabilitation and certified in Ai-Chi. The first session was the introductory session and was not included in the 12-week program. The Ai-Chi program took place in a pool that was 1.40 m deep, with a water temperature of 34 °C (±0.5 °C), and a room temperature of approximately 24 degrees (± 1 °C). The first 10 min of the warm-up period consisted of free limb movements or activities with different pool materials. The Ai-Chi program lasted 20 min and consisted of 16 different movements (out of 19 total). The Ai-Chi exercises, when performed in water at shoulder height with knees slightly bent, using a combination of deep breathing and slow, wide movements of arms, legs, and torso, work on balance, strength, relaxation, flexibility, and breathing. A 15 min water cool-down program (free walking and stretching) was performed after the Ai-Chi program.

#### 2.2.3. Combined Therapy Group

This group received joint aquatic and dry land therapy sessions. The patients (13 people) received alternate dry land therapy sessions (Monday and Wednesday) and aquatic Ai Chi therapy (Tuesday and Thursday), under the same conditions, and the total number of sessions (12 dry therapy sessions and 12 aquatic therapy sessions), as the participants in the control and experimental groups.

### 2.3. Statistical Analysis

Descriptive statistics are reported as the mean ± SD. The normality of the distribution of all variables was assessed using the Shapiro–Wilk statistical test. The effect of the three different rehabilitation protocols was assessed by an analysis of variance of two factors—the first factor was the effectiveness of each of the therapies, and the second factor was the time the effect of the therapy employed in each of the intervention groups was maintained. To determine whether a treatment improves study variable scores compared to another, two-factor ANOVA tests were performed with repeated measurements using the General Linear Model (GLM) procedure. For the effect size, it was considered that, if an eta squared of approximately 0.01 is a small effect, around 0.06 indicates medium effect and above 0.14 is considered a large effect, considering that the points for Phi: 0.1: small effect, 0.3: medium effect and 0.5: large effect. A value of *p* < 0.05 was considered statistically significant. All analyses were performed using the SSPS-23 statistical package (SPSS Inc., Chicago, IL, USA).

## 3. Results

The final study sample comprised 40 patients, of whom 37.5% were women and 62.5% were men, aged between 35–71 years with a mean age of 56.8 years (SD = 15.2). According to the random assignment of treatment groups, 13 patients conformed the combined therapy group, 14 were in the dry land therapy group, and 13 were assigned to the Ai Chi group. There were no significant differences in the demographic and clinical variables between the groups (Table 1)

Table 2 below shows the results obtained in each of the scales used to evaluate the different groups at three different times (before starting therapy, at the end of therapy, and one month after the last session).

**Figure 2:**
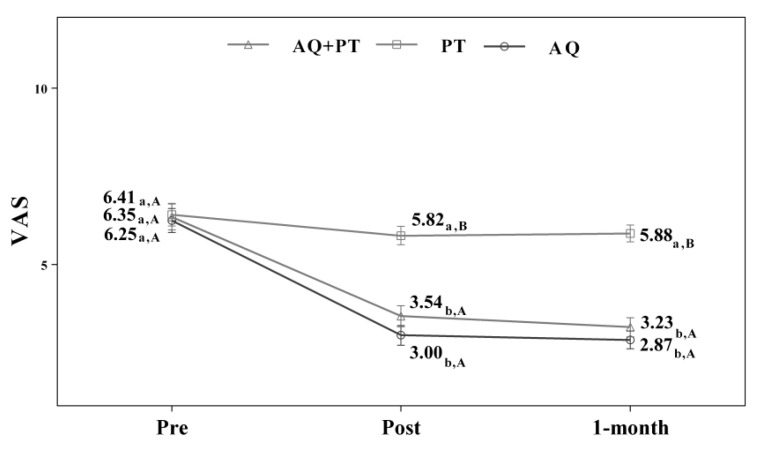
Changes observed in the values of the VAS scale used in the assessment of the participating groups. The bars represent the mean ± SD. AQ + PT group—aquatic therapy and dry land therapy group; PT group—dry land therapy; AQ group—aquatic therapy group; VAS—Visual Analog Scale. a–b—In the same group, different lower-case letters indicate statistically significant differences between time moments (Bonferroni correction). A–B—At the same point in time, different capital letters indicate statistically significant differences between the groups (Bonferroni correction).

**Figure 3:**
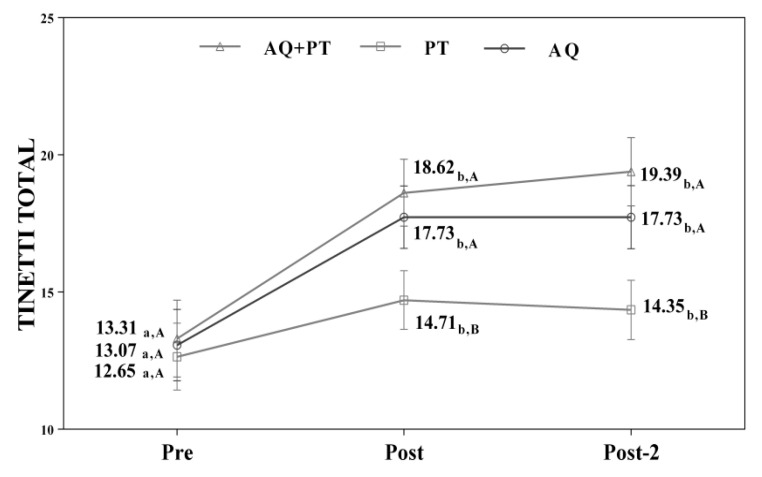
Changes observed in the values of the Tinetti scale (total) used in the assessment of the participating groups. The bars represent the mean ± SD. AQ + PT group—aquatic therapy and dry land therapy group; PT group—dry land therapy; AQ group—aquatic therapy group. a–b—In the same group, different lower-case letters indicate statistically significant differences between time moments (Bonferroni correction). A–B—At the same point in time, different capital letters indicate statistically significant differences between the groups (Bonferroni correction).

**Figure 4:**
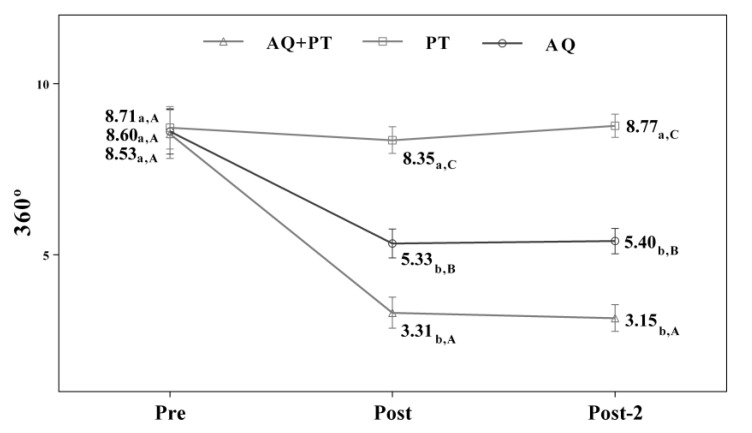
Changes observed in the values of each of the 360° scale used in the assessment of the participating groups. The bars represent the mean ± SD. AQ + PT group—aquatic therapy and dry land therapy group; PT group—dry land therapy; AQ group—aquatic therapy group. a–b—In the same group, different lower-case letters indicate statistically significant differences between time moments (Bonferroni correction). A–B—At the same point in time, different capital letters indicate statistically significant differences between the groups (Bonferroni correction).

**Figure 5:**
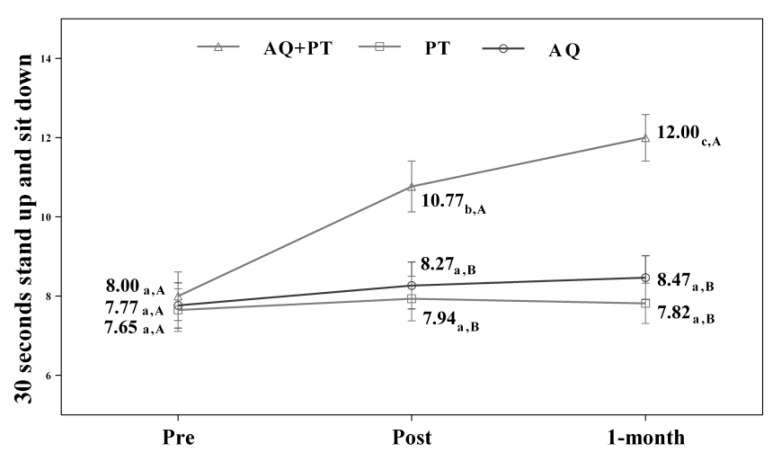
Changes observed in the values of each of 30-s stand-up and sit-down scale used in the assessment of the participating groups. The bars represent the mean ± SD. AQ + PT group—aquatic therapy and dry land therapy group; PT group—dry land therapy; AQ group—aquatic therapy group. a–b—In the same group, different lower-case letters indicate statistically significant differences between time moments (Bonferroni correction). A–B—At the same point in time, different capital letters indicate statistically significant differences between the groups (Bonferroni correction).

Assuming that there were no statistically significant differences in the baseline measurement for any of the variables, diverse outcomes were found in the values obtained by each of the scales and intervention groups. After analyzing the results obtained in the scales used in the assessment, for the main effect of time, there were significant differences in the evolution of the patients independent of the treatment group. The results obtained revealed significant differences between the beginning of the treatment and the results measured at the end, and these differences were maintained one month later. The aquatic therapy and combined therapy groups showed significant improvements at the end of treatment, and these improvements were maintained over time on the VAS pain scale (Figure 2), Tinetti total (Figure 3), the 360-degree rotation (Figure 4), and the 30-s chair stand test (Figure 5). The remaining variables evaluated revealed improvement, although this was not as significant; however, no differences were found between the values obtained in the control group (dry land therapy) throughout the measurements (Figure 2, Figure 3, Figure 4 and Figure 5).

## 4. Discussion

The purpose of this study was to investigate the effect of aquatic exercise on mobility and balance among stroke patients. The results showed that the people in the combined group (dry land + Ai Chi) and the aquatic therapy group were able to reduce the risk of falling. The results revealed that the implementation of low-intensity dynamic movements in water, based on dynamic flexibility, muscle, and balance work, reduces the risk of falls by increasing postural mobility.

Water is a fluid medium, with medium density and viscosity, which reduces the speed of movement, and because of this, when an individual enters a pool with water at waist level, approximately 50% of the weight is reduced, in addition to reducing gravity, in water, the probability of falling decreases by 21–23%. This means that people experience greater mobility in water with greater range of motion [5,8,15,21,22,23,24,25,26].

After reviewing the literature on aquatic therapy in stroke patients, aquatic exercise has been shown to improve motor function and static and dynamic balance. A previous study [27] evaluated the effects of water and land-based jumping training on static balance in patients with chronic stroke. After the intervention, the group that received water therapy significantly improved their static balance compared to the group of patients that received therapy on dry land. In another related study [28], researchers also evaluated the effects of underwater treadmill training, concluding that, compared to the conventional rehabilitation program, treadmill training was no more effective at improving balance skills in comparison to training on dry land. This result may be contradictory to the results obtained in other related studies. Some differences were seen depending on the degree of stroke severity, time since stroke onset, and the specific training exercise protocol. In the study by Zhizhong et al. [29], which also studied the effect of the underwater treadmill for gait retraining in stroke patients, underwater treadmill training was found to improve gait, and Halliwick, and Ai Chi methods, in addition to enabling training on obstacles, were found to benefit the improvement of dynamic and static balance. As in the present study, the combination of an aquatic therapy program with dry land therapy suggests that this treatment protocol could improve functional mobility.

Ai-Chi is a technique that is applied in deep water, with the water at shoulder height and the knees slightly bent; therefore, water resistance is available for all limbs and the torso while practicing Ai-Chi. Coco [30] and Corvillo [31] also reported benefits in muscle strength in patients with multiple sclerosis after aquatic programs, similar to a study by Noh [26] with an aquatic work program in stroke patients for eight weeks. The results obtained by Coco were based a 15-week program, whereas Gehlsen obtained similar results after a 10-week intervention. However, in our study, we obtained similar results over a longer intervention time (12 weeks Ai-Chi). In light of the above results and our findings, this study indicates that Ai-Chi can improve muscle strength in neurological diseases. Our study shows that the beneficial effects obtained have continuity over time (one month after the end of therapy). This suggests that the improvements observed in balance ability are more likely due to optimization of compensatory balance control strategies, such as the strengthening of the ankle and hip strategies on the non-paretic side, improved trunk control, optimization of gait strategies, and an overall adjustment of motor responses to altered sensory input and body dynamics.

Another feature of the Ai-Chi technique is that it includes standing exercises with leg support, such as Tai-Chi on land, which reduces the fear of falling in the aquatic environment. The single-leg stance balance test improved most in the aquatic therapy group (exclusive and combined with dry land), and the muscle strength of the lower limbs also improved, which may have contributed to this finding. Salem et al. [32] found that a five-week program (twice a week) improved balance scores in neurological patients diagnosed with multiple sclerosis. The improvements in the scores obtained by the patients in our study showed a similar tendency to those reported in Salem’s study, even though the pathology was different, however, both were of a neurological nature and had similar deficits. Similarly, in another study investigating the combined effects of Halliwick and Ai-Chi methods on stroke patients, Noh et al. [26] reported that an eight-week program significantly increased the Berg and Tinetti’s Balance Scale scores. In a recent study by Teixeira et al. [33] balance also improved significantly after a 6-week Ai-chi program in older adults, with differences in performance-oriented mobility assessment. A hypothesis demonstrating the improvement in balance in the groups that received part or all of their sessions in an aquatic environment may be due to the increased capacity to support the weight of the trunk and legs, thanks to specific trunk mobility work and lower limb muscle strengthening exercises. In addition, the improvement in balance observed in the aquatic therapy groups (more specifically in the group that received combined therapy) was encouraging as the affected patients could confidently and safely carry out activities of daily living that allow them to sustain their autonomy for longer and in improved conditions.

Another possible cause of the improvement seen among these patients may be due to the temperature of the environment. The temperature of the water in which the activities are performed (no less than 33º) may lead to an increase in skin temperature, peripheral vasodilation, muscle relaxation, and a decrease in pain perception or muscle spasm, thus improving balance [24].

The duration of sessions has been examined by Park, Tripp, and Chu [8,15,24]. In patients with stroke, improvements in balance and gait were shown with 45-min sessions, combining two weekly Halliwick sessions and two other conventional physical therapy sessions [8]. Improvements were also seen in six weekly 35-min sessions of balance and hip, and knee mobilization work 34 or one-hour sessions three times a week [28]. In our study, another option for distributing frequencies and the number of sessions has been shown, which is also positive for obtaining the desired benefits in cardiovascular function, walking speed, balance, and muscle strength.

One of the limitations of our study is the small sample size. A larger randomized controlled clinical trial is needed to validate the benefits reported in our study. In addition, the therapist was not blinded to the exercise group, and although unavoidable, this limitation may introduce bias.

## 5. Conclusions

In conclusion, 12 weeks of aquatic therapy with Ai Chi and combined aquatic therapy with therapy on dry land is effective in improving pain, static and dynamic balance, functional ability and, thus the quality of life in patients with chronic stroke. These improvements may persist for at least one month after completion of the program. These findings support the inclusion of aquatic therapy within the functional rehabilitation exercise protocol of chronic stroke patients and combining conventional therapy (dry land therapy) with activities in an aquatic environment.

## Figures and Tables

**Table 1:** Demographic and clinical characteristics of the study participants.

Characteristics	Treatment			*p*
	AQ + PT Group (n = 13)	PT Group (n = 14)	AQ Group (n = 13)	
Age (years)	53.1 ±11.5	54.6 ±12.1	54.2 ±13.4	0.789
Sex (%)				0.634
Females	5 (38.5)	7 (50.0)	7 (53,8)	
Males	8 (61.5)	7 (50.0)	6 (46.1)	
Previous history (%)				0.709
No	9 (69.3)	10 (71.4)	9 (69.2)	
Yes	4 (30.7)	4 (28.6)	4 (30.7)	
Surgical interventions (%)				0.311
No	4 (31)	7 (50.0)	5 (28.4)	
Yes	9 (69)	7 (50.0)	8 (61.5)	
Medication (%)				0.047
No	4 (30.7)	0 (0)	2 (15.3)	
Yes	9 (69.3)	14 (100)	11 (84.6)	
BMI (kg/m^2^)	26.5 ±2.7	25.8 ±2.9	25.3 ±3.3	0.836
Time since the lesion (years)	6.6 ±3.2	5.7 ±2.6	5.4 ±4.7	0.162

Note: AQ + PT group: aquatic therapy and dry land therapy group/ PT group: dry land therapy/ AQ group: aquatic therapy group. BMI: Body Mass Index.

**Table 2:** Mean values (SD) of the clinical evaluations and intra-subject Generalized Linear Model (MGL) effects.

Rating Scale	Pre	Post	1 Month	Time	Treatment *Time
	Mean ± SD	Mean ± SD	Mean ± SD	F(g.l.); *p*-Value (eta^2^)	F(g.l.); *p*-Value (eta^2^)
VAS_SCALE				F(1.2; 55.7) = 156.82; *p* < 0.001 (0.788)	F(2.9; 55.8) = 24.64; *p* < 0.001 (0.551)
AQ + PT group	6.53 ± 1.1	3.68 ± 1.2	3.38 ± 0.8		
PT group	6.61 ± 1,6	5.91 ± 1.3	5.78 ± 1.2		
AQ group	6.46 ± 1,5	3.11 ± 1.4	2.89 ± 1.0		
TINETTI_BALANCE				F(1.1; 45.2) = 54.81; *p* < 0.001 (0.566)	F(2.2; 45.2) = 4.81; *p* = 0.011 (0.186)
AQ + PT group	8.00 ± 3.4	10.69 ± 2.6	11.15 ± 2.7		
PT group	7.18 ± 2.2	8.18 ± 2.0	8.12 ± 1.9		
AQ group	7.73 ± 2.5	10.47 ± 2.8	10.47 ± 2.8		
TINETTI_GAIT				F(1.2; 48.4) = 76.87; *p* < 0.001 (0.647)	F(2.3; 48.4) = 6.89; *p* = 0.002 (0.247)
AQ + PT group	5.31 ± 3.0	7.92 ± 2.6	8.23 ± 2.5		
PT group	5.47 ± 2.1	6.53 ± 1.7	6.24 ± 1.8		
AQ group	5.33 ± 2.6	7.27 ± 2.5	7.27 ± 2.5		
TINETTI_TOTAL				F(1.1; 45.2) = 77.90; *p* < 0.001 (0.650)	F(2.2; 45.2) = 6.67; *p* = 0.002 (0.241)
AQ + PT group	13.31 ± 4.2	18.62 ± 4.8	19.39 ± 4.9		
PT group	12.65 ± 4.0	14.71 ± 3.3	14.35 ± 3.4		
AQ group	13.07 ± 4.9	17.73 ± 5.1	17.73 ± 5.1		
360°				F(1.2; 50.3) = 49.39; *p* < 0.001 (0.540)	F(2.4; 50.3) = 11.77; *p* < 0.001 (0.359)
AQ + PT group	8.53 ± 2.0	3.31 ± 1.1	3.15 ± 1.0		
PT group	8.71 ± 2.7	8.35 ± 2.3	8.77 ± 1.9		
AQ group	8.60 ± 2.7	5.33 ± 1.0	5.40 ± 1.0		
RIGHT SINGLE-LEG- STANCE-BALANCE				F(1.1;45.4) = 21.19; *p* < 0.001 (0.335)	F(2.2;45.4) = 7.63; *p* = 0.001 (0.266)
AQ + PT group	4.90 ± 3.1	19.31 ± 3.1	21.54 ± 3.7		
PT group	3.88 ± 3.6	4.24 ± 3.1	3.94 ± 3.1		
AQ group	4.80 ± 6.0	10.60 ± 9.0	11.80 ± 8.2		
LEFT SINGLE-LEG- STANCE-BALANCE				F(1.1; 48.0) = 14.21; *p* < 0.001 (0.253)	F(2.3; 48.0) = 5.84; *p* = 0.004 (0.218)
AQ + PT group	3.24 ± 3.5	21.23 ± 2.8	28.62 ± 2.7		
PT group	3.59 ± 3.3	3.77 ± 2.8	3.59 ± 2.8		
AQ group	3.87 ± 4.6	9.73 ± 6.6	11.53 ± 5.2		
CS-30				F(1.6; 65.5) = 84.75; *p* < 0.001 (0.669)	F(3.1; 65.5) = 22.24; *p* < 0.001 (0.514)
AQ + PT group	8.00 ± 1.7	10.77 ± 1.9	12.00 ± 1.8		
PT group	7.65 ± 2.2	7.94 ± 2.2	7.82 ± 2.1		
AQ group	7.77 ± 2.6	8.27 ± 2.7	8.47 ± 2.4		

Note: AQ + PT group—aquatic therapy and dry land therapy group; PT group—dry land therapy; AQ group—aquatic therapy group; CS-30—30 s chair stand test; VAS—Visual Analogue Scale. Eta ^2^—partial Eta squared (effect size); *—Greenhouse–Geisser correction; MGL—Generalized Linear Model.
